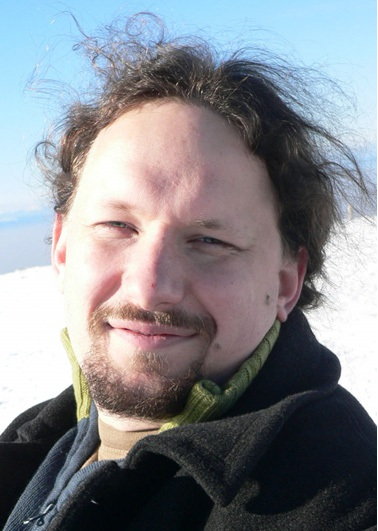# The Mechanism of N‐Heterocyclic Carbene Organocatalysis through a Magnifying Glass

**DOI:** 10.1002/chem.201903021

**Published:** 2020-02-11

**Authors:** Oldamur Hollóczki

**Affiliations:** ^1^ Mulliken Center for Theoretical Chemistry University of Bonn Beringstrasse 4+6 53115 Bonn Germany

**Keywords:** N-heterocyclic carbenes, organocatalysis, reaction mechanism, thiamine, umpolung catalysis

## Abstract

The term “N‐Heterocyclic carbene organocatalysis” is often invoked in organic synthesis for reactions that are catalyzed by different azolium salts in the presence of bases. Although the mechanism of these reactions is considered today evident, a closer look into the details that have been collected throughout the last century reveals that there are many open questions and even contradictions in the field. Emerging new theoretical and experimental results offer solutions to these problems, because they show that through considering alternative reaction mechanisms a more consistent picture on the catalytic process can be obtained. These novel perspectives will be able to extend the scope of the reactions that we call today N‐heterocyclic carbene organocatalysis.

## Graphical abstract

**How does it really work?** The mechanism of N‐heterocyclic carbene organocatalysis has been the subject of debate throughout the last century. Novel results put this old discussion back into focus, revealing more details about these valuable processes. This review summarizes the key elements of the progress, highlighting the arguments that led to the presently accepted mechanistic picture, and underlining the still open conceptual questions.

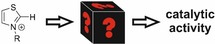

## Introduction

**Figure 1:**
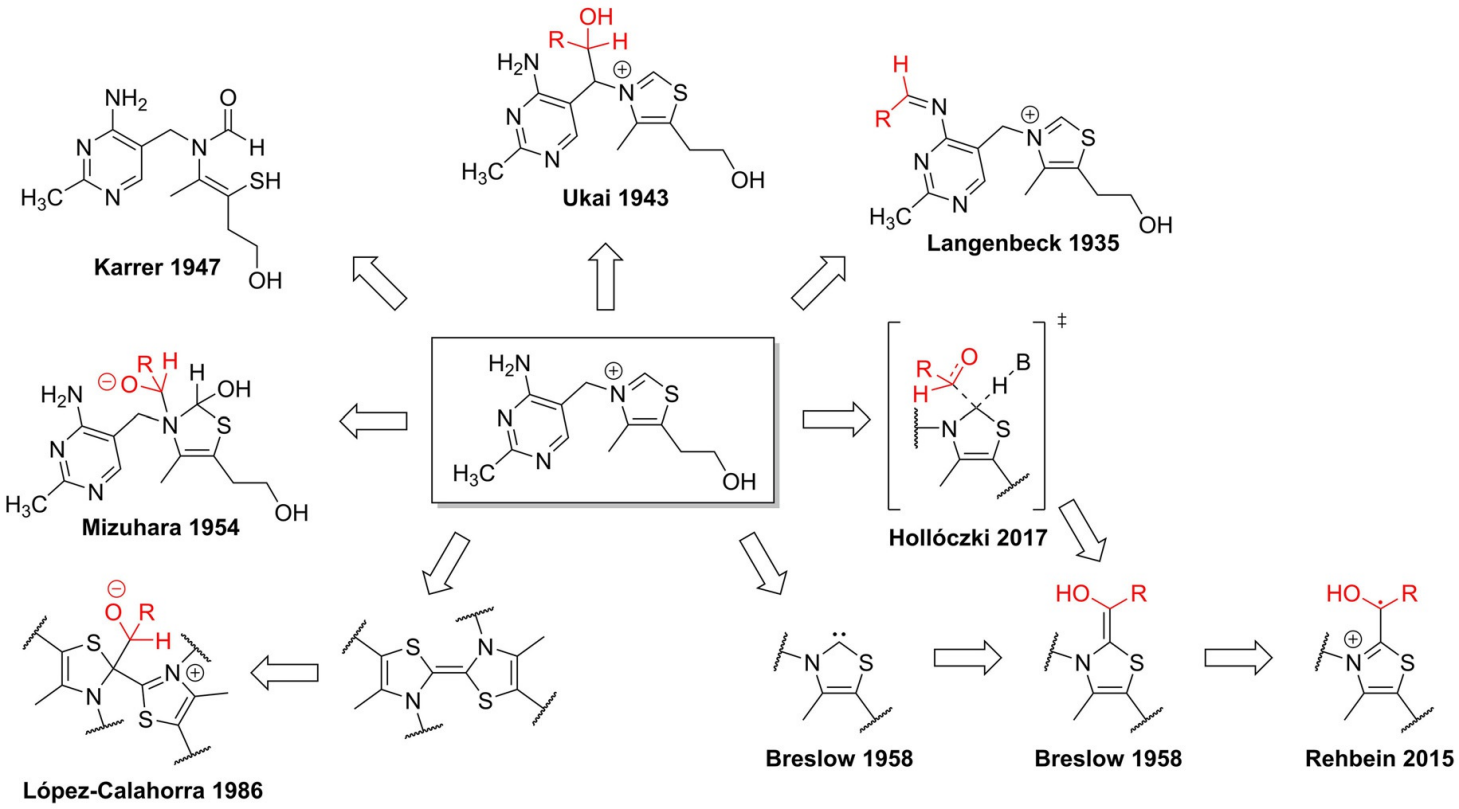
Thiamine, and the key intermediates or transition states of the hypothesized reaction mechanisms of its reaction with various substrates (marked red).

N‐Heterocyclic carbenes (NHCs) define a highly versatile field of chemistry. Significant portion of this knowledge and the corresponding applications grew out of a series of experiments at the end of the 19^th^ century, in which Eijkman observed that rice husk prevents beriberi‐like symptoms of malnutritioned hens.^1^ In the following decades the compound responsible for this effect, thiamine (vitamin B1, Figure 1), was isolated^2^ and its structure was determined, which led to an extensive research on the role of this compound in the human body, and on the mechanism, in which this role is fulfilled. The underlying questions could be answered only through the intensive and interdisciplinary collaboration of biologists and biochemists with organic chemists, who characterized the related enzymes and their function that gave ideas for synthetic applications, and designed model reactions that allowed explaining and even predicting reactions in living organisms.

The first biochemical reaction associated with thiamine was the decarboxylation of pyruvate,^3^ yielding acetaldehyde. However, Green et al. found that carboxylase enzymes from pig hearts did not only decarboxylate pyruvate, but also coupled the product acetaldehyde in vitro to acetoin, which was the first reported benzoin condensation reaction without cyanides.^4^ Independently, Ukai dissolved thiazolium salts in ethanol, and reacted them with benzaldehyde, and he found that—in agreement with Green—benzoin was formed.^5^ A decade later, Horecker^6^, ^8^ and Racker^7^ simultaneously discovered a biochemical reaction of the thiamine‐dependent enzyme transketolase, in which thiamine catalyzes the transfer of a two‐carbon‐atom carbohydrate unit between sugars in a reaction that is chemically analogous to the reactions of Green et al.^4^ and Ukai.^5^ Due to the mutual biochemical and synthetic importance of these C−C coupling reactions, benzoin condensation catalyzed by thiamine and its analogues became the workhorse for later mechanistic investigations.

Stetter recognized the synthetic value of the reactions. By extending the scope of these syntheses, he and then others laid down the fundaments of the field called today “NHC organocatalysis”,^9^ which offers a remarkable portfolio of highly efficient synthetic methods. As a result of these studies, throughout the last century, NHCs and their reactions played a part in the development of biochemistry and medicine, synthetic chemistry, general chemistry and electronic‐structure theory. Although these fields were from the beginning highly intertwined, and built on each other in a synergistic manner, it is important to remember that the initial motivation to go down on this path in science was to understand the biochemical reactions of thiamine and the analogous organocatalytic reactions of NHCs.

In a century of research, the mechanistic picture on these reactions has been continuously refined and extended, and many details of these processes have been revealed. Nonetheless, there has been a multitude of data in literature that does not fit into the general wisdom regarding these reactions, which suggests that our knowledge on these processes is far from complete. Collecting these contradictions is necessary, if a more complete view on these reactions is to be built. To this end, in this critical review the findings that prove or challenge the widely accepted mechanism of NHC‐related organocatalytic reactions are collected, aiming not at giving a full account on these many times reviewed reactions and their applications,^10^, ^11^, ^12^, ^13^, ^14^, ^15^ but rather at focusing on the still open conceptual mechanistic questions.

## Initial Mechanistic Investigations

Given the multiple functionalities in the thiamine molecule, over the decades several proposals had been published for the mechanism of the benzoin condensation (Figure 1). It was suggested^16^, ^17^ that the amino group of thiamine is responsible for the decarboxylation of pyruvate through a Schiff base (imine) formation and a subsequent decarboxylation and hydrolysis. Although multiple model reactions of amines were presented as proof, it was shown that the amino group of thiamine itself was ineffective as a catalyst under the same conditions.^18^ The reaction was also surmised to involve the open‐chain isomer of the thiazolium ring,^19^ but no direct evidence has been presented for the open form being active. In the light of the similarities between alkylpyridinium^20^, ^21^ and alkylthiazolium cations, the methylene bridge of thiamine was also surmised to react with carbonyl compounds.^5^

In his early report, Ukai showed that the benzoin condensation can be catalyzed by thiazolium compounds with a variety of substituents on the nitrogen atom, indicating that the activity of thiazolium salts—and thereby thiamine as well—should be related to the thiazolium ring.^5^ Through using isotopically labeled substrates, further evidence was presented that the thiazolium ring is responsible for the catalytic activity of thiamine.^22^ Breslow recognized that the proton at 2‐position of the thiazolium ring can be exchanged to a deuteron in deuterated methanol.^23^ Thus, he argued that the active species that in fact catalyzes the reactions of thiamine is an NHC, formed by the deprotonation of the thiazolium ring.^24^ Considering that the benzoin condensation is catalyzed by thiazolium salts, he hypothesized that the process responsible for the reaction should be similar to the one cyanide‐catalyzed reaction that had been discovered more than a hundred years earlier by Liebig and Wöhler.^25^ Thus, he adjusted the mechanism established by Lapworth^26^ for the cyanide catalyst, and created the mechanistic picture that is 60 years later still the dominant school of thought for azolium catalyzed benzoin condensations, and was used as a template for designing an array of analogous reactions that comprise the majority of the so‐called NHC organocatalysis, and to explain their action.^10^, ^11^, ^12^, ^13^, ^14^, ^15^, ^27^, ^28^, ^29^, ^30^, ^31^

**Figure 2:**
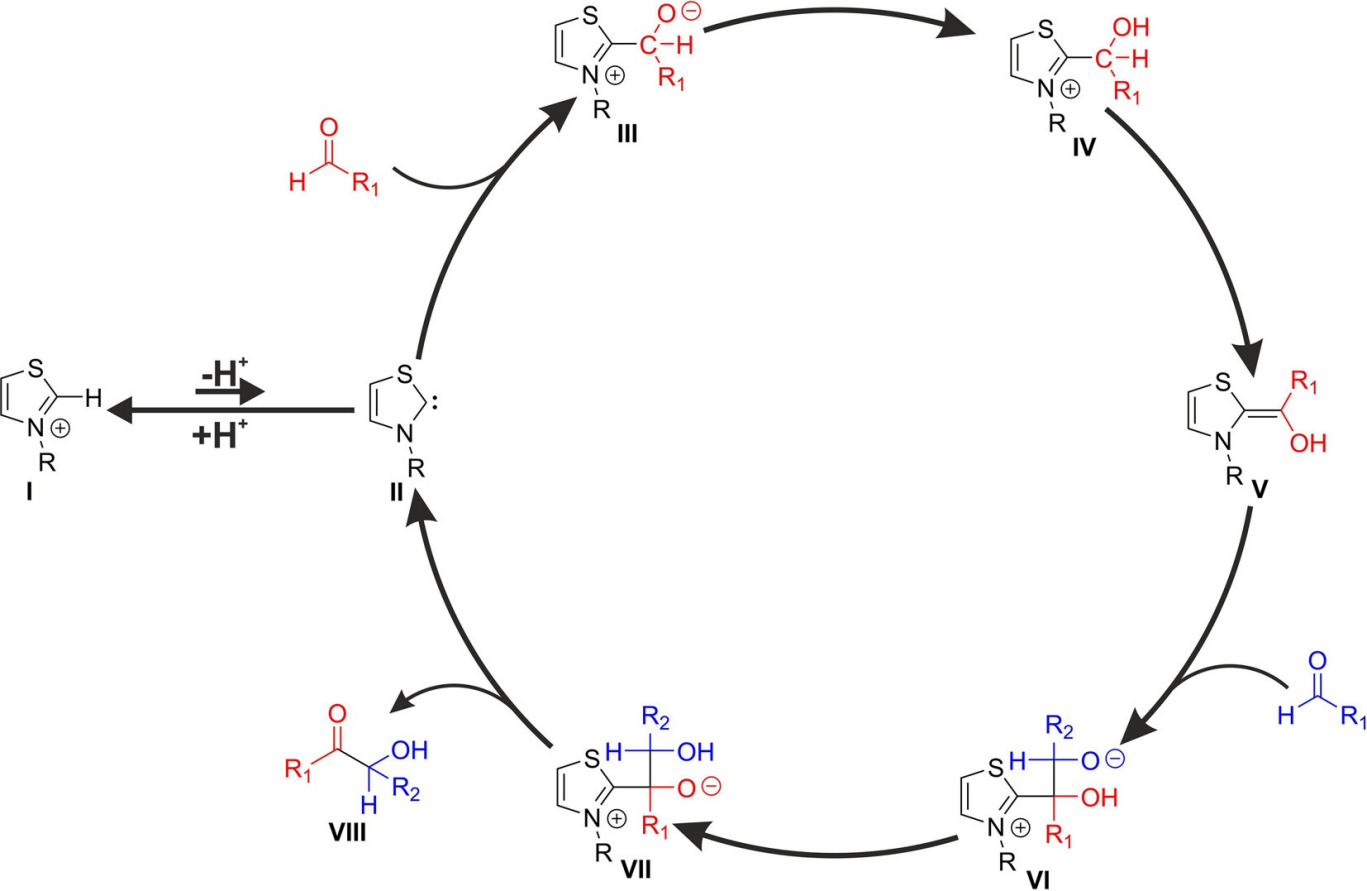
Catalytic cycle of the thiazolium‐catalyzed benzoin condensation as defined by Breslow^24^ used frequently as a paragon for NHC organocatalysis.

In this mechanism,^24^ the initial step is the deprotonation of azolium salt **I** into an NHC **II** (Figure 2). This nucleophilic NHC reacts with the electrophilic substrate (e.g. an aldehyde), and forms an initial (or primary) adduct **III**. Adduct **III** can isomerize into **V** through a protonation/deprotonation mechanism. This structure—nowadays called Breslow intermediate—is another key intermediate of the mechanism, because the fulvenic structure makes its exocyclic double bond polarized in a manner that the electron density shifted away from the ring. This excess of electrons at the exocyclic carbon atom turns this originally electrophilic carbonyl carbon atom of the substrate into a nucleophilic site. Similarly to the “umpolung” in case of the cyanide‐catalyzed benzoin condensation, this polarity change allows an electrophile (e.g. another substrate) to bind to this carbon atom, which makes this reaction valuable for synthesis. Even more importantly, although the benzoin condensation with cyanide only aromatic substrates can be applied, azolium cations can catalyze analogous reactions with aliphatic substrates as well, increasing the scope of the corresponding applications. After the formation of this new bond and a proton transfer, the NHC **II** and the product **VIII** can dissociate, closing the catalytic cycle.

By extending the mechanistic picture above, other NHC‐catalyzed reactions can be explained. With Michael acceptors, Breslow intermediates can yield to give 1,4‐diketones in the Stetter reaction.^27^, ^32^ α,β‐Unsaturated aldehydes form Breslow intermediates with conjugated π‐systems, which—through homoenolate formation—can yield γ‐butyrolactones^30^, ^33^ and β‐lactones^34^ with other aldehydes. Elimination of a benzoate from Breslow intermediates, and a subsequent deprotonation was shown to give azolium enolates,^35^ which are strong nucleophiles^36^ that can be utilized in asymmetric [2+3] cycloaddition reactions.

In support of this mechanism, stable Breslow intermediates^37^, ^38^, ^39^ and analogous structures^38^, ^40^, ^41^ have been detected or synthesized. Recently, a thiazolium salt was tailored for a tandem MS study, which enabled the observation of the actual free NHC intermediate from the evaporated solution, interpreted as a proof for the occurrence of this species in the solution.^42^ Many intermediates of the biochemical processes have been observed as well, being consistent with the model reactions of Breslow.^43^ In the last decades also several theoretical studies have been published, which showed that through this mechanism numerous experimentally observed features of these reactions can be reproduced and explained.^44^, ^45^, ^46^, ^47^, ^48^, ^49^

This mechanism assumes the in situ formation of NHCs in the reaction mixture. During the 1990s, when the “renaissance of carbenes”^50^ was at its high point, Teles et al. showed that not only thiazolium, but also imidazolium and triazolium compounds catalyze these reactions,^51^ presumably with the same reaction mechanism. Accordingly, the community started to exchange the term “thiazolium catalysis” (used by Breslow^24^, ^52^) to “N‐heterocyclic carbene organocatalysis”, which seemed to be a more general term. However, in most of the studies that followed Breslow in further exploring or exploiting the mechanism—including theoretical calculations—the formation of the NHC was considered granted, but it was barely investigated explicitly. In fact, as will be shown below, several studies have been reported that contradict this hypothesis, particularly regarding the involvement of NHCs therein.

## Basicity of N‐Heterocyclic Carbenes

The key to the mechanism above is the acidity of the azolium ring, which allows the formation of the actual NHC catalyst. The earliest estimates for the acid strength of thiamine at this site gave p*K*_a_=12.7,^53^ and p*K*_a_=17–21,^54^, ^55^, ^56^, ^57^ until Washabaugh and Jencks gave exact measurements of p*K*_a_=18.0 for free thiamine in water.^58^ They argued that thiamine must have a p*K*_a_ ≤14, for the formation of the carbene intermediate that would render the carbene formation feasible in the reactions. For the puzzling lower acidity of the compound they gave two alternative explanations. Firstly, it is possible that the enzyme somehow stabilizes the NHC, shifting the acidity of thiamine below the given threshold. This is supported indirectly by earlier data, which showed an acceleration of the catalytic activity of this vitamin by a factor of 10^4^.^54^, ^57^, ^59^ The NHC intermediate was recently also observed within the enzyme,^60^ also in line with this hypothesis. Secondly, they tentatively suggested that this stepwise mechanism that involves the NHC intermediate could be bypassed by an alternative, concerted mechanism, in which the proton transfer and the thiamine–substrate bond formation occurs simultaneously.^58^ However, they rendered this explanation unlikely due to surmised steric considerations.^58^

Although the hypothesis that enzymes change the acid–base equilibrium of thiamine might indeed explain how the reaction can occur through the NHC isomer even with the *pK*_a_ values above, it does not explain the observed high catalytic activity of azolium salts in enzyme‐free organic synthesis. So far three groups of azolium derivatives have been found to be active in organocatalysis: thiazolium, triazolium, and imidazolium salts.^51^ Various derivatives of these catalysts have been found active in the condensation of formaldehyde into different carbohydrates in DMF, with triethylamine as deprotonating agent.^51^ The basicity of these NHC derivatives (i.e., the acidity of their conjugate acids) was in the focus of research in the last decades.^58^, ^61^, ^62^, ^63^, ^64^, ^65^, ^66^, ^67^, ^68^ The strong basicity of imidazol‐2‐ylidenes—p*K*_a_=19–24, depending on the substituent and slightly on the solvent—earned them the title “superbase”. Although thiazol‐2‐ylidenes (p*K*_a_=17–19)^58^, ^61^ and triazol‐5‐ylidenes (p*K*_a_=14.9–17.4)^61^ are somewhat less basic, they are still overwhelmingly more basic than the amine bases that they are deprotonated with (e.g. p*K*_a_=10.65 for trimethylamine^69^). Recently, benzoate derivatives have been also found sufficiently basic to allow NHC organocatalysis.^70^, ^71^ In fact, the presence of the benzoic acid derivative was evidenced in the later steps of these reactions, allowing a dual NHC–Brønsted acid catalysis.^70^, ^71^ Given that the proton transfer from the azolium cation to the benzoate should occur only in a small proportion, it seems likely that the benzoic acid stays associated with the catalyst throughout the following reaction steps. Thus, in other words, NHC catalysis can be performed in a locally acidic environment.^70^, ^71^ Although acid–base theory is one of the most fundamental principles of chemistry, these contradictions have never been thoroughly discussed after the aforementioned considerations of Washabaugh and Jencks.^58^

**Figure 3:**
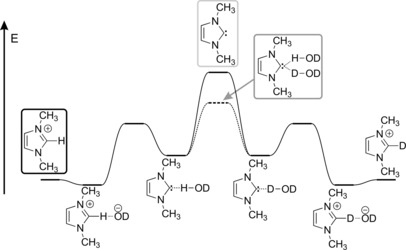
H/D exchange mechanism of azolium cations in D_2_O. The solvent‐exchange step can occur either via the free NHC or the double hydrogen‐bonded structure, both marked with a grey frame.

The high basicity of NHCs makes them also strong hydrogen bond acceptors, a feature that has been suggested first by Wanzlick,^72^ and evidenced later by theoretical calculations^73^, ^74^, ^75^, ^76^, ^77^, ^78^, ^79^ and experiments.^74^, ^80^, ^81^, ^82^, ^83^ This is also in accordance with the observations that solvent rearrangement—that is, the exchange of a hydrogen bond donor at the basic site of the NHC (Figure 3)—is the rate limiting step of H/D exchange reactions of azolium cations. Depending on the NHC and the hydrogen bond donor, the dissociation energy of the hydrogen bonds can be up to even 20 kcal mol^−1^,^75^, ^84^ which is by far stronger than the approximately 5 kcal mol^−1^ value for a water–water hydrogen bond.^85^ This prominent strength should be an obstacle for NHC organocatalysis, because the availability of the lone pair acceptor site of the hydrogen bond is also the cornerstone of the catalyst–substrate bond formation. Thus, if the lone pair is occupied by a hydrogen bond, it should be stabilized against and therefore blocked from undergoing reactions. Considering that the proton transfer from the azolium cation to the base should lead to the formation of a very strong hydrogen bond between the NHC and the protonated base, it is puzzling how carbenes, which are presumably generated in such a small quantity due to their basicity, and then inactivated by the remarkably strong hydrogen bonding, can exhibit any kind of measurable catalytic activity.

## Stable Carbenes

**Figure 4:**
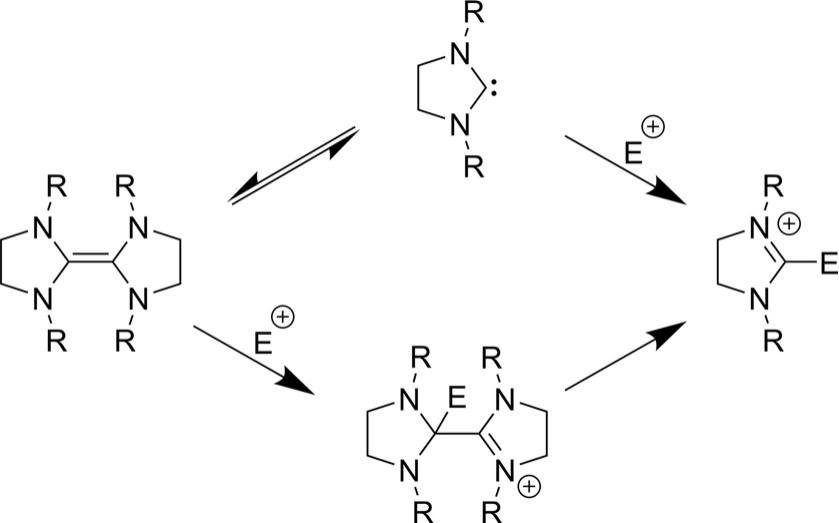
The Wanzlick equilibrium (above),^86^ and an alternative mechanism established by Lemal (below).^92^

**Figure 5:**
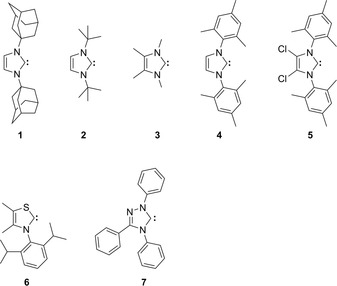
Examples of stable (persistent) N‐heterocyclic carbenes.

The involvement of NHCs in the organocatalytic reactions of azolium salts was supported by the synthesis of free NHCs. Already in the early 1960s, Wanzlick reported that bis(1,3‐diphenylimidazolidin‐2‐ylidene) dissociates into monomers in an (NHC)_2_↔2 NHC equilibrium,^86^, ^87^ and exhibits the chemistry of free NHCs (Figure 4). He also proved that diaminocarbenes and thiazol‐2‐ylidenes are nucleophiles and hence they can react with carbonyl compounds,^72^ seemingly confirming the mechanism established^24^ by Breslow. These findings were strongly corroborated by the synthesis of the first stable NHC 1,3‐diadamantyl‐imidazol‐2‐ylidene **1**,^88^ and later the others 1,3,4‐triphenyl‐1,2,4‐triazol‐5‐ylidene^89^ **7** and 3‐(2,6‐diisopropylphenyl)‐4,5‐dimethylthiazol‐2‐ylidene^90^ **6** (Figure 5). NHC **1** exhibited extraordinary stability under inert atmosphere even at its melting point 240–241 °C,^88^ whereas the 4,5‐dichloro derivative **5** was even identified as “air stable”.^91^

**Figure 6:**
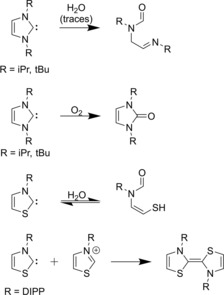
Various decomposition reactions of NHCs.

**Figure 7:**
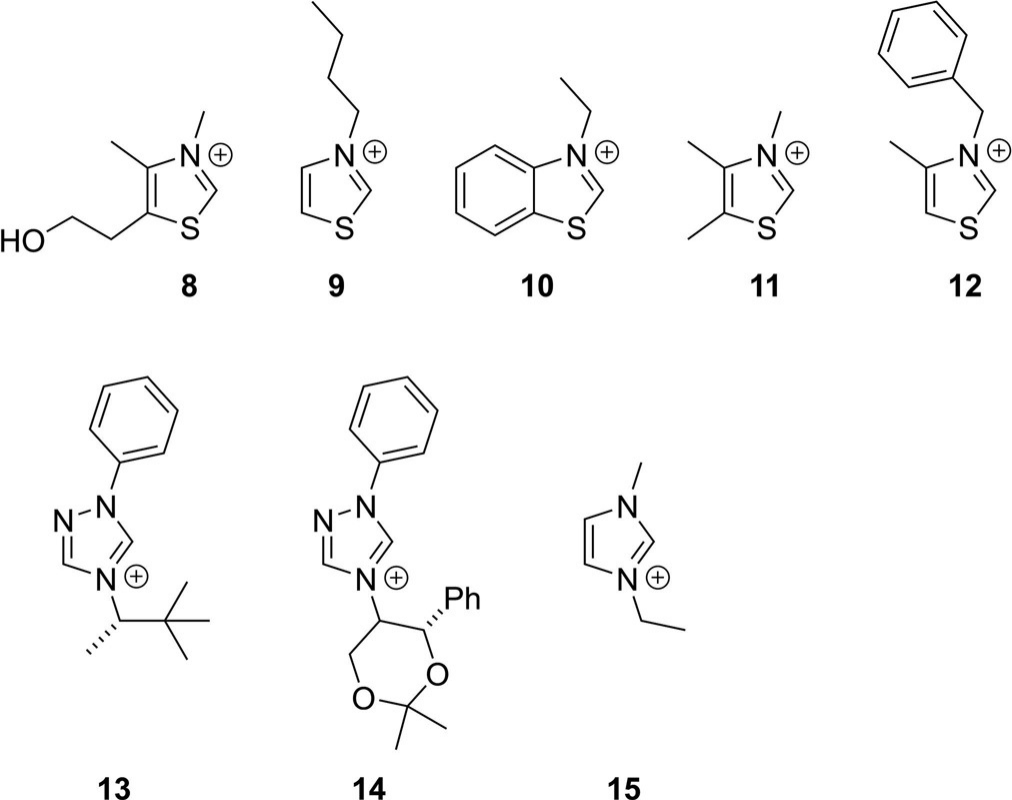
Examples of azolium catalysts employed in NHC organocatalysis.^10^

However, under closer scrutiny these arguments are somewhat less convincing. The successful synthesis of free N‐heterocyclic carbenes in an isolated environment, which is very different from the catalytic mixture, is in fact no direct proof that during the synthesis these species are actually generated. To avoid undesired reactions even under inert atmosphere, stable free NHCs are, except for some imidazol‐2‐ylidene derivatives (e.g., **3**), decorated with bulky substituents. It was shown that the dimerization of thiazol‐ylidenes occurs through the reaction of a thiazolium cation and the corresponding NHC,^93^ and even the bulky 2,6‐diisopropylphenyl substituents of **6** are not enough to fully prevent these side reactions^90^ in the presence of acid traces. These findings raise the question how the in situ generated thiazol‐2‐ylidenes can avoid reacting with the thiazolium catalyst in the reaction mixture of an organocatalytic setup. Furthermore, in the presence of air, almost all hitherto synthesized NHCs react with moisture or oxygen to give various decomposition products,^74^, ^94^ even if the hydrolysis of imidazol‐2‐ylidenes with traces of water appears is sluggish (Figure 6).^74^, ^94^ Despite all this data, most reactions that are called NHC organocatalysis are performed under air,^10^ and often with azolium cations possessing significantly smaller substituents than those mentioned above,^10^ and the introduction of larger substituents into NHCs is merely a way to introduce stereoselectivity (Figure 7).^10^, ^12^

It is, of course, a possible explanation that the concentration or the lifetime of the free NHC is just low enough to avoid these reactions, but high enough to exhibit the desired reactions with reasonable rates. However, to fulfill these two criteria at the same time would mean a lack of robustness for the reactions, and there should be only a narrow basicity range for the reaction media that enables catalytic activity without the decomposition of the catalyst. Given that many different kinds of NHCs are employed in catalysis, each of them with a wide spectrum of bases and solvents, this argument seems unlikely. Following the principle of Occam's razor, a simpler explanation may exist for these contradictions, namely the existence of an alternative mechanism that does not necessitate the presence of free NHCs in the solution.

## Alternative Reaction Mechanisms

Shortly after Wanzlick presented^72^, ^86^, ^87^ the dissociation equilibrium of (NHC)2↔2NHC, Lemal suggested that the NHC‐like reactions occur directly from the dimer, without the involvement of free NHCs (Figure 4).^92^ Based on these findings, López‐Calahorra hypothesized that in the thiazolium catalyzed benzoin condensation this NHC dimer plays the central role as the actual active species. This is supported by the aforementioned propensity of thiazol‐2‐ylidenes to form dimers in the presence acid traces.^90^, ^93^ They established two possible reaction mechanisms, one with the dimer dissociating after reacting with the substrate, resulting in the Breslow intermediate and a free thiazol‐2‐ylidene, from which point the reaction could follow the mechanism of Breslow.^95^ In the other mechanism, the connection between the two thiazolium rings is retained throughout the whole reaction.^95^, ^96^ López‐Calahorra reported that the yields obtained in benzoin condensations by catalysts, in which two thiazolium rings were linked by −(CH_2_)_*n*_− (*n*=2–8) groups through their nitrogen atoms, is highly dependent on the length of the link. They interpreted this dependency as a direct proof for the mechanism involving the NHC dimer.^97^ However, isotope‐labeling experiments corroborated the original mechanism by Breslow, and thereby the involvement of the dimer in the reaction was questioned,^93^ and the related enzyme structures also showed no possibility for the formation of thiamine dimers in biochemical reactions.^93^, ^98^ Breslow showed that the reaction kinetics was first‐order in thiazolium salts, and the transition state of the rate‐limiting step contains two benzaldehyde molecules and a single thiazolium.^99^ López‐Calahorra presented kinetic data that he rationalized as second order in thiazolium salts,^100^ contradicting the earlier measurements. Bofill presented a computational study,^101^, ^102^ finding that the reaction occurs through a biradical mechanism with the NHC dimer. In turn Breslow re‐analyzed the data of López‐Calahorra, showing that it is in fact first order in the thiazolium catalyst, and that their interpretation was erroneous.^52^ Thereafter, this mechanism was not discussed any further. Moreover, later theoretical calculations show that many NHCs do not form dimers,^103^ which renders organocatalytic reactions through these structures as a general mechanism somewhat dubious.

Through molecular dynamics simulations we have observed that the exchange of a solvent molecule, which is in hydrogen bond with the NHC, does not necessitate the formation of the free NHC in the solution.^77^, ^79^ Instead, the single lone pair can accommodate a second hydrogen bond donor, allowing for an associative exchange,^77^, ^79^ which can facilitate the hydrogen bond dynamics—thus the solvent exchange—of NHCs (Figure 3). We assumed that if the capacity of NHC lone pairs to serve as multiple interaction sites is a general feature, it may also allow a concerted reaction mechanism for the related organocatalytic reactions, as was suggested (and immediately rejected) earlier.^58^

**Figure 8:**
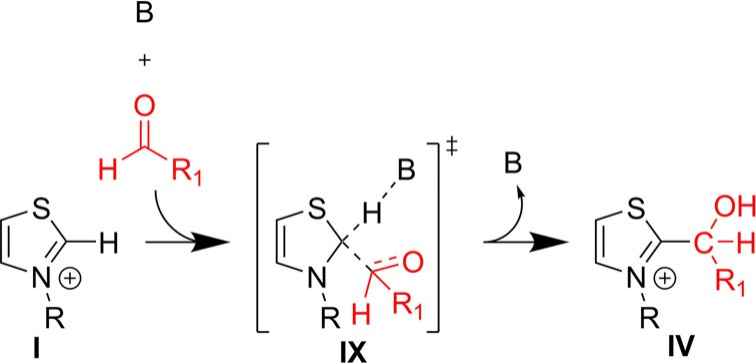
Alternative reaction mechanism for the initial step of the reaction between azolium cations and aldehydes, through a single elementary step, without the formation of free carbenes (see Figure 2).^104^

We could identify two mechanisms for the reaction between azolium cations and aldehydes in the presence of trimethylamine base.^104^ The first, dissociative or stepwise reaction mechanism follows the mechanism as suggested by Breslow (Figure 2), including the explicit formation of a free NHC in solution. In the second, associative or concerted mechanism (Figure 8), the association of all components occurs first, forming an initial cluster. Within this cluster, the catalyst–substrate bond can form within a single elementary step through a proton transfer from the cation to the amine base and a simultaneous C−C bond formation between the ring carbon atom at the active site of the catalyst, and the substrate, yielding directly the protonated adduct of the dissociative mechanism.^104^ Through this path, the reaction can occur despite the large basicity difference between the NHCs and the bases without the formation of the free carbene, which is therefore not present in the solution, and cannot show any decomposition reactions depicted in Figure 6. The activation energies, enthalpies and Gibbs free energies indicate an overwhelming dominance of approximately 20–30 kcal mol^−1^ for the associative mechanisms for all nine combinations of the three azolium cations and three aldehydes that were investigated. Using continuum solvent models did not change this general conclusion, although polar media decreased the differences in barriers.^104^ Interestingly, changing the base from amines to an acetate anion decreases the advantage of the associative mechanism,^105^ which has implications for the chemistry of ionic liquids (for an excellent review see Ref. 106).

The barrier in the dissociative mechanism originates largely from the difference in basicity between the NHC and the amine. In the associative mechanism, the transfer of the proton plays a role, therefore the basicity of the NHC could have an effect on the barrier. Indeed, the barriers of the two paths showed a common trend,^104^ which also explains why this possibility has been overlooked previously. In other words, a less acidic azolium cation should have a slower reaction through both paths, in agreement with the general qualitative trends in the experiments.^51^ The first qualitative measurements of such processes, which aimed at observing these two reaction mechanisms, were performed by Rico del Cerro et al.^107^ They calculated the rate constants for the two paths of H/D exchange reactions of imidazolium salts through DFT calculations and found that the experimental values compare better to the associative mechanism,^107^ confirming our computational results. These findings, and the availability of the associative reaction mechanism provide a consistent picture on the reaction mechanism of the organocatalytic reactions catalyzed by azolium derivatives.

The practical importance of this seemingly subtle difference can be recognized, if one considers the role of dissociative and associative mechanisms of nucleophilic substitution reactions, S_N_1 and S_N_2 processes, in chemistry. It is well known that the presence of the leaving group in the rate‐limiting step of S_N_2 reactions has a structure‐directing effect, which may be exploited to improve stereoselectivities. Similarly, the presence of the (protonated) base in **IX** (Figure 8) may allow influence in the formation of the initial substrate–catalyst bond, which might be important in the presence of multiple substrates. Recent studies on structure‐directing effects by the presence of the protonated base at the later steps of NHC organocatalysis seem to underscore this hypothesis.^70^, ^71^

**Figure 9:**
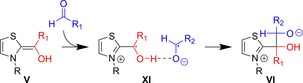
Alternative reaction mechanism for the C−C coupling step of the benzoin condensation through a single electron transfer (Figure 2).^113^, ^114^

Regarding the later steps of the catalytic cycle also further details have been revealed. Already in the early work of Bofill it was recognized^101^ that radical and biradical pathways may play a role in organocatalytic reactions by azolium salts, even if the actual mechanism he suggested has been disproven.^52^ It was also shown that Breslow intermediates can be oxidized, to generate a radical species,^108^ a feature that has been shown to play a role in biochemical reactions,^109^ and has been exploited in the last decade to perform redox catalysis with NHCs.^31^, ^110^, ^111^ Rehbein observed EPR signals in a benzoin condensation setup, with the exclusion of oxygen. The radical was observed at the onset of the reaction, and it was evidenced that the formation of the species in question requires both the Breslow intermediate and the aldehyde. Kinetic isotope effects did not only confirm this finding, but were also found similar to those observed for the overall reaction earlier,^112^ which could be explained through the rate‐limiting step of the radical formation and the overall reaction being identical.^113^ These findings were consistent with a single‐electron transfer from the Breslow intermediate to the aldehyde substrate to give **XI** as an intermediate before the C−C bond formation. In a subsequent study, it was shown that the quantum chemically calculated and experimentally measured kinetics compare well,^114^ which provides further proof for this alternative mechanism. Accordingly, the mechanism established by Breslow can be extended by a radical pathway (Figure 9). However, in a follow‐up study Regnier et al. showed evidence that the one‐electron oxidation of the Breslow intermediates leads to a subsequent deprotonation of these species, yielding acylium radicals, which are therefore more likely to be present in the reaction than **XI**.^115^ The authors also point out that even if the formation of radicals has been observed in the benzoin reactions, it has not been yet proven that these radicals are actual genuine intermediates of these reactions.^115^ These considerations make it clear that further studies are necessary to assess the importance of radical formation in these processes.

## Summary and Outlook

Since the discovery of thiamine, a lot of hypotheses have been published regarding the biochemical and organocatalytic activity of this vitamin and its analogue azolium cations. Many of these possible ideas have been proven wrong since then, defining an evolution of the mechanistic picture for these processes. Although nowadays the overall mechanism of Breslow with the formation of NHC intermediates in solution is accepted, and on some examples it has been directly proven by a multitude of studies, there are still results that point to other possible paths for these processes. The two main current directions in this regard are a mechanism, which bypasses the formation of free NHCs in the solution, and an electron‐transfer process between the Breslow intermediate and aldehyde substrates, resulting in the formation of radicals. Both of these mechanisms need more research in terms of NHC rings, substituents, bases, counterions, and solvents, to evaluate under which conditions are they dominant over the classical catalytic cycle as defined by Breslow. The possibility of avoiding carbenes in the so‐called “NHC organocatalysis” raises the question, if the community should change the name describing these reactions to “azolium catalysis”, which fits better to the term “thiazolium catalysis” used by Breslow even in 1996,^52^ while also describing more accurately the actual catalytic processes.

## Conflict of interest

The author declares no conflict of interest.

## Biographical Information

*Dr. Hollóczki received his PhD degree in the Budapest University of Technology and Economics. After receiving an Alexander von Humboldt Fellowship for Postdoctoral Researchers*, *he joined the group of Prof. Barbara Kirchner in 2012 at the University of Leipzig. He is currently working on his habilitation at the University of Bonn in the topics of N‐heterocyclic carbenes and sustainable chemistry. In 2018 he was awarded the Prize of the Association of German University Professors in Chemistry*.